# Framing UN Human Rights Discourses on Climate Change: The Concept of Vulnerability and its Relation to the Concepts of Inequality and Discrimination

**DOI:** 10.1007/s11196-023-10092-1

**Published:** 2024-01-12

**Authors:** Monika Mayrhofer

**Affiliations:** 1 Department of Applied Sociology of Law and Criminology, University of Innsbruck, Innsbruck, Austria; 2 University of Innsbruck, Vienna, Austria

**Keywords:** Concept of vulnerability, Climate change, Human rights, United Nations, Equality, Discrimination, Frame-analysis

## Abstract

The concept of vulnerability is widely used in human rights policy documents, reports, and case law focusing on the impacts of climate change on human rights. In academic discussions, the concept, however, has also sparked a discussion on its benefits and challenges for the advancement of human rights, especially concerning the principles of equality and non-discrimination. This article aims at contributing to this debate from a frame-analytical perspective. In social sciences, frame-analysis is a form of discourse analysis which focuses on the consequences of different concepts for legal, political, and social discussions and norms. With the example of selected UN documents on different human rights issues in the context of climate change, the article, firstly, analyzes whether and how the concept of vulnerability is defined in the documents and why it is used in the documents. Secondly, it is elaborated to which individuals and groups the concept is applied. Thirdly, it is discussed how vulnerability is conceptualized in relation to or in distinction to the concepts of inequality and discrimination. In a further section it is analyzed what narratives are mobilized by the frame of vulnerability. The article concludes that from a discourse-analytical perspective the frame of vulnerability mobilizes problematic narratives which has gendered and racialized implications for those labeled vulnerable.

## Introduction

United Nations (UN) institutions, in particular human rights bodies, have increasingly focused on the impact of climate change on human rights. One of the most recent contributions to this topic is a report by the UN Secretary-General on ‘The impacts of climate change on the human rights of people in vulnerable situations’, which was published on 6 May 2022. The title of this report refers to a core concept that is continuously used by human rights bodies and actors when discussing the consequences of climate change for the enjoyment of human rights: vulnerability. Vulnerability is generally a popular concept in the context of human rights. Although the concept is not mentioned in international human rights instruments it is explicitly applied in human rights policy documents, reports, and jurisprudence. Turner has pointed out, that the term vulnerability originates from the Latin word ‘wound’ therefore recognizing the ‘obviously corporeal dimension of existence’ and the ‘fact’ that human beings are ‘ontologically vulnerable and insecure’ [1, pp. 26–28]. Although this meaning seems plausible at first glance, it is often less clear what the concept means when applied in the political and legal context. A glance at the literature as well as at policy documents reveals that the concept is frequently used to refer to many different thematic dimensions and levels of application. In the human rights context, the concept is often applied to individuals, groups, and communities whose rights are directly or structurally in danger of being violated, who are marginalized or who are in a precarious situation, so-called ‘vulnerable groups’ or ‘vulnerable individuals’.

The concept of vulnerability has stimulated a discussion on its benefits and challenges for the advancement of human rights,^1^ especially concerning the principles of equality and non-discrimination [7–20]. Some refer to the potential promises of the concept such as being a more substantial basis for equality, taking into consideration the ‘natural’ dimension of human beings in the human rights context or universalizing rights entitlements as the vulnerable subject is proposed to be a more universal figure as the liberal subject. In addition, the recognition of vulnerability is perceived as a ‘condition for the respect of human dignity’ [21], the concept is assumed to allow for getting rid of identity categories and the norm of the liberal subject by replacing it with the ‘vulnerable’ subject. In doing so, it is assumed to overcome the limits of formal equality which is based on the ideal conception of the liberal, independent, and rational subject and it is supposed to concentrate on the structures of society [11, 18, 22]. More skeptical researchers, however, stress the ‘deficit-orientated nature of the term and its link with stigma’ [7, p. 319],^2^ which ‘tends to emphasise people’s weaknesses and limitations, and is in danger of showing people as passive and incapable of bringing about change’ [24, p. 13]. They criticize the vagueness of the concept which undermines ‘its promise as a conceptual frame to understand and challenge systematic inequalities’ [25, p. 266] and makes it 'difficult to reconcile with ensuring equal protection of human rights’ [26, p. 205]. Furthermore, critical scholars highlight the reduction of the concept to specific ‘vulnerable groups’ which is often not only a stereotypical representation of these groups but may also hamper the objectives of human rights. For example, the labeling of specific groups as vulnerable may have negative consequences for groups or individuals excluded from the concept [13] or may be complicit in practices of essentialism, stigmatization, and paternalism [18, 20, 27–29]. It was also argued that the concept is a problematic entry point into politics as the ‘vulnerable citizen is in certain respects the antithesis of proper citizenship’ [27, p. 670]. In addition, it was pointed out that as a result of vulnerability reasoning ‘dynamics of dominance and inequality shift towards questions of feeling’ [25, p. 274] and that ‘it prevents equality’ [30, pp. 155–156].

The increasing utilization of the concept of vulnerability therefore raises questions about the meanings and implications of the concept in the field of human rights in general and its relation to the concepts of equality and non-discrimination in particular. A study published in 2011 on the application of the concept of vulnerability by the UN Committee on Economic, Social and Cultural Rights (CESCR) pointed out that the Committee does neither offer a clear-cut conception or definition of vulnerability or related terminology nor provide a comprehensive listing of groups qualifying for this designation. The study also suggested that the Committee had then recently dropped vulnerability terminology and substituted it with the terms disadvantaged and marginalized [31, pp. 723–724]. In UN human rights documents focusing on climate change in general and climate change-related mobilities in particular, it cannot be established that the concept is used less. However, a shift in these documents could be observed: Instead of vulnerable groups and persons, the formulation of groups, people or individuals in vulnerable situations is increasingly used, which is also reflected by the document mentioned at the beginning of this article.

Therefore, although the concept of vulnerability is used widely in UN human rights discourse, the substance, theoretical underpinnings, and objectives of the concept are often not well-defined and its relationship to the concepts of inequality and discrimination is not clear. This article analyzes how vulnerability is conceptualized and used in UN documents focusing on human rights and climate change including those with a particular emphasis on climate change-related mobility. Concerning the latter, it has been argued that people moving in the context of climate change are often framed as vulnerable, helpless, and passive victims who need protection [32]. Human rights approaches are said to play a crucial role in perpetuating this victim-protection narrative [32, p. 254], [33, 34]. The discursive emphasis of this frame is on the suffering and abuses (of rights) of specific persons and groups and on policies and regulations that protect affected individuals and groups. The concept of vulnerability is assumed to be an important part of this narrative [32, p. 109].

Therefore, this article will systematically analyze documents published by UN human rights institutions and bodies that focus partly or entirely on climate change. In doing so, it will address the following research questions:

- How is vulnerability defined in UN documents on climate change and human rights? What is the substance, what are the different dimensions of the concept and to which situations is it applied? What are the objectives, motivations, and legitimation for introducing and using the concept in the analyzed documents?
- To which groups and persons does the concept refer? Does the shift from vulnerable groups to people in vulnerable situations change the narrative?
- How does the concept of vulnerability used in the analyzed documents differ from, contradict or overlap with the concepts of (in)equality and non-discrimination? What is the role of the concept of vulnerability concerning the guarantee of human rights, especially concerning the containment of discrimination and the enhancement of equality? What are the consequences of the concept concerning structural dimensions of inequality and indirect discrimination?
- What are the narratives and stories associated with these concepts in the documents? What are the associations with the frame of vulnerability that are discernible in the analyzed documents? How does the concept depict persons framed as vulnerable?

### Methodological and Theoretical Approach

The documents selected for this analysis were ‘sampled purposively’ based on being particularly relevant and informative concerning the topic of interest [35, p. 211], [36, p. 211], i.e. the topic and leading questions of this article. In a first step, documents published online by UN human rights institutions and bodies were screened, and those, which focus either entirely on human rights issues and state obligations in the context of climate change or which address the issue partly in a substantive manner,^3^ were selected for analysis (in total 67 documents). The selected texts comprise reports, policy briefs or documents, case law, declarations, resolutions, recommendations, or comments and were published, for example, by the UN Human Rights Council (HRC), special procedure mechanism, the Office of the United Nations High Commissioner for Human Rights (OHCHR), but also by treaty-based human rights bodies. In a second step, the documents were uploaded into a Qualitative Data Analysis (QDA) program (MAXQDA), coded according to a specific framework, and qualitatively analyzed concerning their insights regarding the research questions. The initial coding framework was developed based on the research questions (deductive coding) and extended and refined by additional codes derived during the coding process (inductive coding). In the last step (the analytical phase), themes were developed based on the codes and thematically grouped and coded extracts were interpreted in the light of the research question [37, pp. 56–64].

To grasp the implications of the concept of vulnerability in the context of the human rights discourse on climate change, a frame-analytical approach was used. This theoretical approach is particularly relevant for Sect. 6, where the stories and narratives associated with the frame of vulnerability, which are discernable in the documents, will be discussed. Frame analysis is a ‘variation of discourse analysis’ [38, p. 6]. On a very basic level, frame analysis highlights the importance of language in legal, political, and social processes and structures and the consequences of using different concepts, words, and arguments for legal, political, and social discussions and dynamics. Frame analysis has been applied by a wide range of academic disciplines, including, for example, linguistics, social movement research, media studies, migration studies, climate change research, gender studies, and public policy analysis [32, 39–49]. Although not as widely used as in the social sciences, frame analysis is also applied to analyze legal discourses [50–54]. Before elaborating on the details of frame analysis that are relevant to this study, I would like to briefly discuss what is meant by the term ‘concept’, since vulnerability first of all is understood as a concept in this article. A concept is conceived in its very basic sense, which means it is seen as ‘a general idea or notion (…); a mental representation of the essential or typical properties of something, considered without regard to the peculiar properties of any specific instance or example. (…) the meaning that is realized by a word or expression.’[54] From a social science point of view, concepts are important in many contexts, including social, political, legal, and academic contexts. ‘Concepts are the way that we make sense of the social world. They are labels that we give to aspects of the social world that seem to have common features that strike us as significant.’ [55, pp. 8–9] Lakoff and Johnson emphasize the importance of metaphors in our conceptual system; they explicitly point out that by metaphor they mean a metaphorical concept. ‘The essence of metaphor is understanding and experiencing one kind of thing in terms of another’. [45, p. 9] They argue ‘that the human conceptual system is metaphorically structured and defined.’[45, p. 6] Furthermore, they illustrate that metaphorical concepts have powerful consequences; they have, as they put it, ‘the power to define reality. They do this through a coherent network of entailments that highlight some features of reality and hide others.’ [45 , p. 115] This understanding is also important for the present study. Vulnerability, which means wound and the possibility of being wounded, is widely used in the analyzed documents as a metaphor, that means as a metaphorical concept, since the literal meaning of the word is an injury to the body, which is transferred to many non-somatic issues in the documents. Metaphors or metaphorical concepts also play an important role in frame analysis.

Frame analysis aims at scrutinizing different meanings and substances, underlying ‘narratives’ and ‘structures of belief, perception and appreciation’ [47], which are influential in policy and legal processes and documents. Frames are assumed to be selective as they highlight ‘some aspects of a perceived reality and make them more salient in a communication text, in such a way as to promote a particular problem definition, causal interpretation, moral evaluation, and/or treatment recommendation’ [44, p. 52].

‘For purposes of communicating about that framing, the features that are selected for attention have to be named. Such policy naming at times invokes metaphors: Concepts whose meaning(s) in other situations is (are) known and understood, such that their use in this situation (typically without conscious intent) makes what is going on clearer.’ [48, p. 99] Thus, frames indicate how a social situation or problem is named and defined, which features are selected for attention by choosing specific ‘metaphors, catchphrases and other condensing symbols’ [56, p. 152], and which assumptions, narratives, and stories are thereby promoted and mobilized. Frames ‘construct particular meanings concerning issues by their patterns of emphasis, interpretation, and exclusion’ [57, p. 217]. A metaphor, such as vulnerability, used as (part of) a frame ‘triggers a larger network of associations’ [53, p. 339] and, thus, enables and mobilizes specific stories or narratives. This is also the case in the legal context. Not only do metaphors play an important role in legal language [50], ‘frames in the legal sphere operate in a similar fashion to the public opinion realm’ [58, p. 618]. Thus, the frame-analytical approach applied in this research aims at giving insights into the narrative consequences of the metaphor of vulnerability in UN human rights documents on climate change and which features are emphasized and which are concealed by applying the concept of vulnerability in these documents.

### Structure

This article starts by discussing the emergence of climate change as a UN human rights issue and the use of the concept of vulnerability in this context. In doing so, the paper gives an overview of the selected documents and the prevalence of the concept of vulnerability in these documents (Sect. 2). In the third section, the paper analyzes whether and how vulnerability is defined in the documents, what thematic dimensions the concept refers to, and whether the documents offer implicit definitions in cases where no explicit definitions can be found in the documents. In addition, this section discusses whether the documents provide an explanation, of why the concept is used and the added value of using vulnerability in the context of human rights and climate change. As the concept of vulnerability is often used to refer to specific groups and individuals, the fourth section gives an overview of which groups and individuals are frequently labeled as vulnerable and discusses whether the shift to the referring to persons or groups in a vulnerable situation instead of vulnerable groups and persons also makes a substantial difference. This is followed by an elaboration on the conceptualization of vulnerability compared to the concepts of inequality and non-discrimination as indicated in the analyzed documents (Sect. 5). Section 6 analyzes which implications vulnerability has when we understand the concept from a frame-analytical perspective. It is thus analyzed which stories about persons, groups, and communities are invoked when the metaphorical concept of vulnerability (the wound) is attached to these persons and groups. It is discussed which features and narratives are emphasized in the vulnerability debate on human rights in the context of climate change and which are neglected. In the last section, the most important insights of the analysis are summarized and the conclusions presented.

## Climate Change as a Human Rights Issue—Overview of Documents

Environmental issues including climate change were put on the human rights agenda relatively late. The so-called Stockholm Declaration, which was adopted at the United Nations Conference on the Human Environment in 1972 and which is considered the ‘foundation of international environmental law’ [59, p. 35], makes the first connection between environmental degradation and human rights. Although the Action Plan for the Human Environment, which was adopted at the same conference, refers to (human-made) climate change, neither the declaration nor the Action Plan mentions the term vulnerability.

Only in 2008, the HRC adopted its first resolution (Resolution 7/23) that explicitly focuses on human rights and climate change. In this document, the Committee voiced its concern ‘that climate change poses an immediate and far-reaching threat to people and communities around the world and has implications for the full enjoyment of human rights’ and recognized that ‘the world’s poor are especially *vulnerable* to the effects of climate change, in particular those concentrated in high-risk areas, and also tend to have more limited adaptation capacities’ [60]. Resolution 7/23 also requested the OHCHR to carry out a detailed analytical study of the relationship between climate change and human rights. The *Report of the Office of the United Nations High Commissioner for Human Rights on the relationship between climate change and human rights* was drafted based on a consultation process with States, intergovernmental and non-governmental organizations, national human rights institutions, and individual experts. The report was adopted on 15 January 2009 and outlines the main aspects of the relationship between climate change and human rights. The concept of vulnerability is used 30 times throughout the document, particularly in relation to ‘vulnerable groups’ with a focus on women, children, and indigenous people [61]. Since then, the HRC has adopted many resolutions [62–72] and published several reports and other documents focusing on climate change and human rights. All resolutions contain a reference to vulnerability, often in the form of a recognition that ‘the adverse effects of climate change will be felt most acutely by those segments of the population that are already in vulnerable situations owing to factors such as geography, poverty, gender, age, indigenous or minority status and disability’ [62].

A specific focus of UN documents has been the impact of climate change on different forms of human mobility and the human rights of persons migrating or displaced in the context of climate change and disasters. Already the 2009 report published by the Office of the United Nations High Commissioner, which was mentioned above, contains a section on displacement. Also in 2009, the *Report of the Representative of the Secretary-General on the Human Rights of Internally Displaced Persons, Walter Kälin,* and the Addendum of the report on *Protection of Internally Displaced Persons in Situations of Natural Disasters* was published [73, 74]. This report was followed by many other reports of different UN bodies and stakeholders focusing either entirely or partly on the issue of human rights of climate-mobile persons. In 2011, the former Special Rapporteur on the human rights of migrants, Jorge Bustamante, submitted a report to the HRC, which outlined, inter alia, possible themes for further study [75]. One of the two topics mentioned in this section was ‘Migration in the context of climate change’. The successor of Jorge Bustamante, François Crépeau dedicated the thematic section of the 2012 Report of the Special Rapporteur on the human rights of migrants to the impacts of climate change and some of its consequences for migration [76]. Two other important reports by the United Nations High Commissioner for Human Rights and the OHCHR focusing on climate change-related mobility were published in 2018: the report on *The Slow onset effects of climate change and human rights protection for cross-border migrants* [77] and the report *Addressing human rights protection gaps in the context of migration and displacement of persons across international borders resulting from the adverse effects of climate change and supporting the adaptation and mitigation plans of developing countries to bridge the protection gaps* [78]. In July 2020, the Report of the Special Rapporteur on the human rights of internally displaced persons, Cecilia Jimenez-Damary, was published [79]. The report examines ‘internal displacement in the context of the slow-onset adverse effects of climate change’. The last report with a focus on mobility that was considered for this study is the 2022 Report of the Special Rapporteur on the human rights of migrants. The report ‘examines the human rights situation of migrants, especially women, children, indigenous peoples, minorities and other groups in specific vulnerable situations, affected by the adverse effects of climate change’ [80]. All these documents make use of the term vulnerable or vulnerability, some of them extensively. For example, the 2018 report on slow onset effects of climate change, which is also a rather extensive document with about 60 pages, mentions vulnerable or vulnerability 111 times.

UN human rights bodies have repeatedly worked towards integrating human rights standards in international climate policies and action.^4^ For example, in the run-up to the Conference of the States in Paris in 2015 (COP 21), the HRC organized a full-day panel discussion with representatives from UN Member States, intergovernmental organizations, civil society organizations, and academia. The results of the panel discussion including key messages on human rights and climate change were submitted to COP 21 by the OHCHR. The messages emphasize that climate change ‘will disproportionately affect individuals, groups, and peoples in vulnerable situations including, women, children, older persons, indigenous peoples, minorities, migrants, rural workers, persons with disabilities and the poor.’ [81] In addition, the OHCHR published specific *Key Messages on the issue of human rights, climate change, and migration* highlighting that policies and negotiations on climate change and migration should ‘Protect the human rights of people who are in particularly vulnerable situations’ [82]. In November 2022, the OHCHR together with the Center for International Environmental Law (CIEL) published a Toolkit for Practitioners on *Integrating Human Rights in Nationally Determined Contributions (NDCs)* stating that NDCs ‘should include information about efforts to protect the rights of those in particularly vulnerable situations from the adverse effects of climate change and ensure that they are the primary beneficiaries of climate action.’ [83]

Different (human rights) bodies and actors have increasingly analyzed the impact of climate change on different topics but also on specific individuals and groups. In August 2009, the General Assembly adopted the *Report of the Special Rapporteur on adequate housing as a component of the right to an adequate standard of living, and on the right to non-discrimination in this context*. The report focuses on ‘the impact of climate change on the fulfilment of the right to adequate housing, especially in respect of how climate change exacerbates existing vulnerabilities’ [84, p. 3]. Other studies concentrate, on the relationship between climate change and the full and effective enjoyment of the rights of the child (2017) [85], on climate change and poverty (2019) [86], on gender-responsive climate action (2019) [87], on the rights of persons with disabilities in the context of climate change (2020) [88] and on the rights of older persons in the context of climate change (2021) [89]. In May 2022, the Report of the Secretary-General on ‘The Impacts of climate change on the human rights of people in vulnerable situation’ was released. In June 2023, the Human Rights Council adopted its latest report on the *Adverse impact of climate change on the full realization of the right to food* [90].

As the first treaty-monitoring body, the Committee on the Elimination of Discrimination against Women (CEDAW Committee) adopted *General Recommendation No. 37 on Gender-related dimensions of disaster risk reduction in the context of climate change* (GR 37) in February 2018 [91]. In 2018, UN Special Rapporteur on Human Rights and the Environment John H. Knox published the *Framework Principles on Human Rights and the Environment* which define ‘the basic obligations of States under human rights law as they relate to the enjoyment of a safe, clean, healthy and sustainable environment.’ [92] Also these documents use the concept of vulnerability, including, for example, Framework Principle 14, which lays down that ‘States should take additional measures to protect the rights of those who are most vulnerable to, or at particular risk from, environmental harm, taking into account their needs, risks and capacities.’ [93] Among the most recent documents included in this study is *Draft General comment No. 27 (202x) Children’s rights and the environment with a special focus on climate change*, which was adopted by the Committee on the Rights of the Child in May 2023.^5^

## Definitions of Vulnerability, Different Dimensions, and Objectives of the Concept

In the documents analyzed for this study, vulnerability is predominantly used without providing a (clear-cut) definition. In general, the provision of a definition should ensure that there is a common understanding of the meaning of a term or concept. A ‘good’ definition ‘tries to point out the features that are essential to the designation of things as members of the relevant group’ and applies ‘to exactly the same things as the term being defined, no more and no less’ [94]. Furthermore, a definition makes it possible to distinguish one issue from another. The explicit specification of a definition in a document may also indicate that there was a reflection on concepts and approaches used. Among the documents reviewed only a few documents provide explicit guidance on how vulnerability is understood or defined. In documents, where definitions of vulnerability are indicated or referred to – oftentimes not explicitly –, they tend to be very broad and general and, thus, they usually fail to indicate the delimitations of the concept and to provide an unambiguous understanding of vulnerability.

The report *The Slow onset effects of climate change and human rights protection for cross-border migrants*, is one of the very few documents, that gives guidance on how the concept is understood. The document contains the following paragraph:

‘This study adopts an understanding of vulnerability that is focused on a person’s relative ability to effectively exercise their human rights. […] vulnerability is understood as both ‘situational’ and ‘personal’. […] Increased vulnerability also means that an individual is likely to have less adaptive capacity—or ability to adjust or respond to the impacts of climate change.’ [77, Para. 52]

Several points are noteworthy in this understanding of vulnerability: Firstly, as a definition it is quite broad and vague and the ‘focus’ on a person’s ability to exercise their human rights is not a specification of the meaning of the concept. Secondly, in this definition, the vulnerable person is understood as the ‘problem’. It is the individual or person’s ability or lack of capacity which is the focus of vulnerability. This is a significant difference compared to definitions of discrimination, where it is the less favorable treatment of a person on specific grounds [95] or the ‘distinction, exclusion, restriction or preference’ on specific grounds ‘which has the purpose or effect of nullifying or impairing the recognition, enjoyment or exercise by all persons, on an equal footing, of all rights and freedoms’ [96] which is defined as the problem. That means in the first instance, the affected individuals themselves and their deficits are conceived as the problem; in the second instance, it is actions and structures that have a detrimental effect on certain people that are identified as the problem. Thirdly, the reference to an ‘increased’ vulnerability suggests that there are gradations in terms of the degree of vulnerability but it is not made explicit against which standard of non-increased vulnerability this is measured. Another example is the *Framework principles on human rights and the environment*, which lay down that ‘[p]ersons may be vulnerable because they are unusually susceptible to certain types of environmental harm, or because they are denied their human rights, or both.’ [93, Para. 40] Although this quote does not exclusively define the affected person as the problem, as it also refers to the denial of human rights, similar to the definition mentioned above, it includes an understanding of the vulnerable person as the deviation of an inherent norm as they are assumed to be *unusually* susceptible to harm. Again, the ‘usual’ standard is not made explicit. In doing so, the norm the definition implicitly refers to is rendered invisible.^6^

The documents point to many different factors that influence vulnerability such as ‘poor health and malnutrition’, ‘a low adaptive capacity’ [61, Paras 32–33], a broad range of ‘societal factors’ such as marginalization, exclusion, discrimination, poverty, limited ability to participated in political life, inadequate participation processes or ‘access to justice’, [76, Paras 27, 78] ‘poverty, gender, age, disability, geography and cultural or ethical background’ [93] and ‘climate change’ [77]. Some documents indicate that exposure to (environmental) harm is a critical feature of vulnerability, some even seem to equate vulnerability and exposure [86, 88, 93, 97].

Besides different factors influencing vulnerability, the concept is used to refer to many different dimensions and contexts: The documents, for example, mention ‘vulnerabilities of persons affected by sudden-onset and slow-onset natural disasters’, ‘increased risk of vulnerabilities throughout the migration cycle’, ‘workplace-related vulnerabilities’ [98, Para. 18 (k), 20 (d) and 23 (d)], ‘food and livelihood vulnerability’, ‘economic vulnerability’, and ‘vulnerability in changes of in rainfall’ [77 Paras 97, 108, 129], ‘vulnerable households’ [78, Para. 60], ‘particular vulnerabilities of food production systems’ [99], or ‘vulnerable to water stress’ [86, Para. 7].

The concept of vulnerability is not only used with regard to individuals and groups it is also frequently applied to refer to locations, communities, regions, countries, and systems. Countries and regions, which are usually labeled as vulnerable, are developing and least-developed countries, African countries, South Asian countries, rural areas, low-lying coastal areas, and small-island States. Thus, the documents convey a picture of an uneven geography of vulnerability, where the concept is preliminary used to refer to regions and countries of the Global South.

The documents do not provide an explanation what are the objectives of the concept of vulnerability and why the concept is introduced and used. It is also not explicitly clarified, what is the added value of using the concept. For example, concerning climate mobility, the ‘causes’ of vulnerability are indicated in the following quote:

‘’Migrants’ vulnerabilities are often created or exacerbated by increasing barriers to international migration, which include its criminalization; migration policies based on deterrence; border restrictions; restrictions on migrants’ access to labour markets in destination countries; and a lack of regular migration pathways, including for work at all skill levels, education, family unity and humanitarian needs. […] As a result, transit can be precarious for irregular migrants, borders are difficult to cross safely, and those with less means to pay for safer transport often face dangerous journeys.’ [77, Para. 52]^7^

The quote describes a list of ‘barriers to international migration’ and deduces thereof the creation or exacerbation of migrant’s vulnerability. The end of the quote, however, lists concrete problematic results of these barriers. It is not clear what is the objective and the added value of the general reference to ‘migrant’s vulnerability’ at the beginning of the quote as it would have been sufficient to describe the concrete problematic results barriers to international migration have for migrants. The same applies to other definitions, mentioned above. For example, the study which ‘adopts an understanding of vulnerability that is focused on a person’s relative ability to effectively exercise their human rights’ [77] or the Framework principles which derive a person’s vulnerability ‘because they are denied their human rights’ [94, Para. 40]. Both contributions could directly focus on the exercise or denial of human rights, which UN human rights bodies have always done, but now they frame the issue as vulnerability without explaining the objective and added value of the use of the concept.^8^

## Vulnerable Groups and Individuals

In the documents, the concept of vulnerability is often used to refer to specific groups and individuals. Increasingly, not the phrase vulnerable groups is used but the term groups or individuals in situations of vulnerability or persons in vulnerable situations.

Persons and groups, frequently labeled as vulnerable in the documents are (different sub-groups of) women (and girls), children, migrants, refugees, disabled, older persons, indigenous people, and the poor. Persons and groups identified as vulnerable occasionally or sometimes are, for example, adolescents, ill persons and persons with health-related problems, survivors of sexual exploitation, abuse and violence, minorities, stateless persons, family, LGBTIQ + , peasants and rural communities, ‘people of African descent’ and persons with HIV/AIDS. The 2009 *Report of the Office of the United Nations High Commissioner for Human Rights on the relationship between climate change and human rights* focuses in a specific section ‘on factors determining vulnerability to climate change for women, children and indigenous peoples.’ [61, Para. 44] Typical quotes are the following examples:

‘Within countries, existing vulnerabilities are exacerbated by the effects of climate change. Groups such as children, women, the elderly and persons with disabilities are often particularly vulnerable to the adverse effects of climate change on the enjoyment of their human rights.’ [61, Para. 94]

‘While an increasing number of persons are affected by and/or displaced as a consequence of a natural disaster, all too often insufficient attention is paid to the multiple human rights challenges they may face. The most vulnerable groups of society – including the poor, marginalized minorities, female- and child-headed households, chronically ill persons, persons with disabilities and older people without family support – suffer the most from the negative effects of natural hazards due to their weakened mitigation and adaptation capacities.’ [74, Para. 25]

Although some groups are identified more often to be vulnerable, it is not clear, what qualifies a certain group as being vulnerable. There is a considerable variety of different groups as well as sub-groups framed as vulnerable listed in the documents. Thus, there is neither a recognizable system in the categorization of groups as vulnerable nor do the analyzed documents provide a denotative definition of vulnerable groups. Yet, although there is considerable arbitrariness as to which groups are designated as vulnerable, there are also striking patterns. The most striking one is that women and different ‘sub-groups’ of women (such as female migrant workers, mothers, and female-headed households) are most frequently labelled as vulnerable. None of the analyzed documents explicitly refer only to men and boys as a vulnerable group.^9^ The same is true for other ‘groups’, while disabled, poor, indigenous, or ill persons are frequently framed as (particularly) vulnerable, able-bodied, rich, non-indigenous, or healthy persons are not explicitly labeled as vulnerable at all.

Increasingly also the formulation of persons in vulnerable situations is used in UN human rights documents, for example, the following recurring text module:

‘while these implications affect individuals and communities around the world, the effects of climate change will be felt most acutely by those segments of the population who are already in vulnerable situations owing to factors such as geography, poverty, gender, age, indigenous or minority status and disability’ [62]

Does this suggest that there might be a shift from pre-determined vulnerable groups to a more nuanced and inclusive conception of people in vulnerable situations according to different inequality categories? The above-mentioned 2022 report on *The impacts of climate change on the human rights of people in vulnerable situations* exclusively uses the phrase ‘people in vulnerable situations’ as indicated by the title. The document does not explain why this phrase was preferred in drafting the document. The preference for this formulation may be due to the desire to avoid attaching the concept of vulnerability to certain groups. In the concept note to the panel discussion leading up to the drafting of the report, there seems to be some awareness of problematic dimensions of the concept as the note explicitly affirms that ‘no one is inherently vulnerable’ [101, p. 2]. However, the concept of vulnerability is nevertheless used as a central reference point and neither is there a clear and explicit definition of the concept nor is there an abandonment of group-focused approaches. From paragraph 4 of the report, it can be assumed that the phrase ‘people in vulnerable situations’ is used to refer to people ‘who are disproportionately at risk from the adverse impacts of climate change’ [97, 97

## Vulnerability and the Concepts of (In) Equality and (Non)-Discrimination

As already pointed out in the beginning, there has been a considerable discussion on how vulnerability is interrelated with, differing from, overlapping with, or even contradicting the concepts of equality and non-discrimination, which are two fundamental and (legally) well-developed and -defined principles of international human rights law.^10^ This section will focus on how vulnerability is conceptualized in relation or distinction to the concepts of equality and non-discrimination. In contrast to the rights to equality and non-discrimination, vulnerability is not mentioned by international human rights law. The analyzed documents indicate, that in most cases vulnerability is not used as a synonym for inequality and discrimination as these terms in the texts are not used in substitution to each other but in addition to each other. For example, documents refer to ‘gender-specific discrimination and vulnerabilities’ [109], ‘pre-existing vulnerabilities and patterns of discrimination’ [74], or ‘existing inequalities and vulnerabilities’ [79]. The Framework principles on human rights and the environment contain one principle dedicated to prohibiting discrimination and ensuring equality (Framework principle 3) and another principle focusing on measures to protect the rights of those who are most vulnerable to environmental harm (Framework principle 14) [93]. As has already been pointed out in Sect. 3, definitions of vulnerability provided by the documents, if any, have a different focus than, for example, definitions of discrimination laid down in international law.

In the UN documents analyzed for this article inequality and discrimination are frequently understood as factors that lead to increased vulnerability. For example, a Report by the United Nations High Commissioner for Human Rights published in 2018 says that vulnerability ‘can result from multiple and intersecting forms of discrimination, inequality and structural and societal dynamics that lead to diminished and unequal levels of power and enjoyment of rights’ [78, Para. 14].^11^

Discrimination and inequality are understood as ‘underlying causes’ [61] or the ‘root causes’ [93, 97], as exacerbating factors [61] or as factors that result in or increase vulnerability [77, 84, 87]. A direct causality between discrimination/inequality and vulnerability is established in many of the UN human rights documents. However, the relationship is also described vice versa [77] or even as a spiral where inequality and discrimination lead to vulnerability and vulnerability leads to more inequality and discrimination:

‘While the impacts of slow onset events are indiscriminate, those already in vulnerable situations are at the greatest risk of suffering human rights harms as a result of their adverse effects. […] These effects will disproportionately impact people already in vulnerable situations due to their ‘geography, poverty, gender, age, indigenous or minority status, national or social origin, birth or other status and disability’. […] For these reasons, or a combination of these reasons, some will also experience discrimination and are at increased risk of human rights violations and abuses before they move, during their journey, and at destination. […] These experiences can create or worsen vulnerable situations for migrants.’ [77, Para. 51]

This circular movement is indicated in several analyzed documents. The starting point for change and action indicated in some documents is addressing inequality or discrimination:

‘Since climate change disproportionately affects the rights of persons living in vulnerable situations, the principles of equality and non-discrimination are particularly relevant to climate actions […].’ [84, Para. 45]

A further notable aspect is, that the terms vulnerable or vulnerability are often accompanied by adjectives such as ‘especially vulnerable’ or ‘particularly vulnerable’ [74, 84, 85, 91, 110], ‘most vulnerable’ [76, 77, 91, 93, 76, 111, 91

A recurring policy demand of the analyzed documents is to give vulnerable persons and groups and people in vulnerable situations priority treatment or particular attention. For example, one document emphasizes that measures ‘shall be offered in a non-discriminatory manner, priority being given only on the basis of specific vulnerability and need’ [74, 61, 93], ‘giving priority to protecting vulnerable individuals and communities’ [61, 77, 112]. This raises the question of whether the priority treatment is comparable with the concept of specific measures (also called ‘positive action’ or ‘temporary special measures’) which is mentioned and defined by international human rights law and instruments. Such ‘specific measures imply a preferential treatment of a person’ on specific grounds ‘to address historic and/or systematic/systemic exclusions from the benefits of exercising rights.’ [106] Although there is repeated reference to priority treatment in vulnerability approaches, the notion of priority treatment or attention is vague and unclear. Contrary to ‘temporary special measures’ which have to fulfill a list of criteria in order not to be considered unlawful discrimination,^12^ the notion of priority treatment in the context of vulnerability approaches is not linked to any criteria in the documents reviewed for this study.

## Narratives Mobilized by the Frame of Vulnerability

In this section, it will be analyzed what narrative consequences the frame of vulnerability has and which aspects are highlighted, when the metaphor of a wound is applied in human rights documents on climate change. The narrative frequently invoked in the documents is that of lists and chains of (potential or actual) harm, abuses, and sufferings faced by vulnerable individuals and groups. Vulnerability is a concept, which is associated with many problematic and adverse adjectives and situations. It is a catch-all phrase that seems to stand for a broad range of adversity. Recurring narratives that are attributed to vulnerable persons, groups, and communities are, firstly, many situations of poor health such as diseases, illnesses, malnutrition but also death. For example, documents stress the particular vulnerabilities of older persons, including older women and older persons with disability or persons in vulnerable situations ‘exposed by the implications of climate change, including their increased susceptibility to diseases, heat stress, […] and reduced physical, emotional […] resilience’ [67, 70]. Concerning ‘vulnerabilities’ of women and girls it is, for instance, emphasized ‘that unequal food systems disproportionately affect women and girls, making them more vulnerable to food insecurity and malnutrition, which is exacerbated, inter alia, by climate change, environmental degradation, and disasters’ [114] or that ‘pregnancy is a period of increased vulnerability to a wide range of environmental hazards, including extreme heat and infectious diseases such as malaria, foodborne infections, and influenza.’[115] Concerning the ‘complex interrelationship between climate change, health, mortality, and migration’ it is pointed out that ‘[m]igration as a result of the negative effects of climate change can create situations of vulnerability that adversely impact health and may lead to mortality.’[116]

A second narrative association which is widespread in the analyzed documents is the connotation of vulnerable persons and groups as victims of different forms of violence, conflict, and other related security issues. For example, it is pointed out that ‘[u]nfortunately migrants are facing increasing intolerance and are becoming more vulnerable to potential racist or xenophobic outbreaks of violence, or they may fall prey to criminal traffickers and smugglers.’ [75] Another example that refers to women and girls reads as follows:

‘women and girls are particularly vulnerable to the adverse effects of climate change and at higher risk of violence during displacement. […] they […] are exposed to a higher risk of sexual and gender-based violence, forced labour, exploitation, abuse and trafficking in persons.’ [79]

The last part of the quote indicates another frequent narrative associated with vulnerable individuals and groups, that is their ‘vulnerability to’ exploitation and abuse^13^ and the repeated emphasis that they are suffering (the most), struggling, or that they are at risk, in some sort of crisis or face challenges:

‘The most vulnerable groups of society […] suffer the most from the negative effects of natural hazards due to their weakened mitigation and adaptation capacities.’ [74, Para. 25]

‘[…] those already in vulnerable situations are at the greatest risk of suffering human rights harms as a result of their adverse effects.’ [77, Para. 5]

‘Slow-onset events, such as sea level rise or desertification, might hit a community that is already struggling to cope with the effects of armed conflict and is therefore more vulnerable to disasters.’ [79, Para. 5]

‘Poor communities can be especially vulnerable, in particular those concentrated in unplanned and unserviced settlements within urban areas, which tend to be built on hazardous sites and to be susceptible to a number of climate change-related disasters. Living in a situation of poverty and exclusion, they lack adequate resources to protect themselves. Climate change-related effects aggravate existing risks and vulnerabilities.’ [117, Para. 16]

Vulnerability is used when referring to precariousness, threat, danger, lack, and other adjectives and nouns that refer to different forms of distress, adversity, deprivations, and hardships. Individuals and groups framed as vulnerable are portrayed as passive (potential or actual) victims or persons with *special* or *particular* needs and who require protection, special attention, priority treatment, and assistance (see also quotes above):

‘States must also protect groups in particularly vulnerable situations from the adverse effects of climate change, disasters and related displacement.’ [79, Para. 42]

‘Climate change adaptation efforts should give priority to the needs of the most vulnerable and start by identifying the measures to be introduced for their protection.’ [117, Para. 74]

‘[…] especially vulnerable persons among the displaced such as children, expectant mothers, mothers with young children, female heads of household, persons with disabilities, persons who are seriously ill or injured and older persons are entitled to protection and assistance required by their condition and to treatment which takes into account their special needs.’ [74]

The reference to vulnerability is often formulated as being a characteristic or feature of a specific person or group. It is something, which is an essential part of their being and which defines them. It is not formulated as something that happens to them or what others do to them, but rather what they are.^14^ For example, ‘particularly vulnerable are those’ [61, 61, 79, 77], migrants are ‘more vulnerable to potential racist or xenophobic outbreaks of violence’ [75], or ‘[g]roups such as children, women, the elderly and persons with disabilities are often particularly vulnerable to the adverse effects of climate change on the enjoyment of their human rights.’ [61, Para. 94] The vulnerability framing not only entails problematic and hierarchic association of the helpless, weak, wounded victim with special needs who requires protection, vulnerability conceptually^15^ leaves out the perpetrator.^16^ The perpetrators of climate change are not mentioned at all in the documents. The focus of the vulnerability framing is on the victims. It is them who are vulnerable to violence, discrimination, abuse, impacts of climate change, and other adversities. The vulnerable person or group is conceptualized as the problem, who needs to be addressed and protected, and whose ‘resilience and adaptive capacities’ have to be supported.^17^ Their needs are labelled as ‘special’ or ‘particular’, thus, implicitly referring to an invisible standard of those (men, able-bodied persons, wealthy, middle-aged persons) who have ‘normal’ needs so that they are not even mentioned as needs at all.

In some of the analyzed documents, it is apparent that there is an ongoing discussion on whether the concept of vulnerability is contributing to the stereotypical categorization of individuals and groups. This is, for example, the case in *General recommendation No. 37 (2018) on the gender-related dimensions of disaster risk reduction in the context of climate change*, where it is pointed out that.

‘[t]he categorization of women and girls as passive “vulnerable groups” in need of protection from the impacts of disasters is a negative gender stereotype that fails to recognize the important contributions of women in the areas of disaster risk reduction, post-disaster management and climate change mitigation and adaptation strategies.’[91]

In another document, it is pointed out that ‘[t]hose disproportionately affected by climate change — including migrants — are not inherently vulnerable and do not necessarily lack resilience or agency. They should not be treated as victims.’ [78, Para. 22]^18^ From a frame-analytical perspective, it must be pointed out that the negation of a frame does not mean that it thereby loses its effect. The opposite is the case. ‘Every negation of a frame activates that frame. Activating a frame means to […] strengthen it.’ [120, p. 84] That means, avoiding problematic effects of a specific frame, in this case, the frame of the metaphor wound which is associated with narratives of illnesses, diseases, harm, threat, risk, violence, suffering, victimhood, special treatment and protection, incapability, weakness and many other situations of adversity, would need to refrain from using this concept in the first place and choose different concepts that enable more empowering narratives.

## Discussion and Conclusions

UN human rights institutions and bodies often use the concept of vulnerability when discussing the impacts of climate change on human rights. However, it is still disputed whether the concept is beneficial for the advancement of human rights, in particular concerning the rights to equality and non-discrimination. This article aimed to contribute to this debate by analyzing how the concept is used in documents (such as reports, studies, and case law) focusing on different human rights issues and topics in the context of climate change which were published by UN institutions. A frame-analytical approach, which pays particular attention to the narratives, conceptual connotations, and meanings and their implications in a discursive setting was used as a theoretical lens. The following points can be concluded from the analysis:

Although the concept is used very frequently, it is hardly ever explicitly defined nor is there any reflection what is the added value of the concept. When definitions are indicated they are rather broad and it is not clear why the concept is necessary as the definition refers to issues that were always the focus of UN human rights institutions and bodies (e.g. the denial of human rights to certain individuals and groups of persons) but which are now framed as their vulnerabilities. The definitions – as well as the narratives mobilized by the concept – suggest that, first and foremost, the vulnerable persons themselves are categorically defined as the problem and not actions and structures that are harmful to them. This is in contrast to non-discrimination and equality approaches, which do not, also not conceptually, focus on the deficit of a person but on problematic actions against and structure between persons. Furthermore, definitions but also narratives mobilized by the frame of vulnerability suggest that the concept is based on an implicit understanding, which conceives person and groups labeled as ‘particularly’, ‘most’, ‘more’, or ‘especially’ vulnerable (e.g. female, poor, mobile, old, young, indigenous, black, sick/ill, disabled, living in/coming from ‘developing’ countries) as a deviation from an inherent norm (e.g. male, wealthy, middle-aged, sedentary, healthy, able-bodied, living in industrialized countries), which is also often referred to as the liberal subject inherent in the human rights project. The vulnerability frame, thus, does not remove the liberal subject, it is rather rendered invisible and unproblematized. The inherent standard of the liberal subject in human rights law and policies and its racialized and gendered implications have been repeatedly criticized.^19^ Yet, particularly in the context of climate change lifestyles attributed to the liberal subject have contributed most to the problem in the first place and, thus, should be problematized and categorically included in the problem definition. Furthermore, this inherent understanding of ‘norm and deviation’ the vulnerability discourse is based on also suggests an inferiority of persons marked as vulnerable, which is also contrary to equality and non-discrimination principles laid down in human rights law.^20^

The analyzed documents indicate that vulnerability is not used as a synonym for inequality or discrimination, although a relation between these concepts is suggested. Inequality and discrimination are described as factors that result in increased vulnerability in the context of climate change-related impacts and that, in turn, may facilitate more discrimination and increased inequality. The recurring demand voiced in the documents that vulnerable persons should be given priority treatment or particular attention is a matter of concern insofar as, due to a lack of definition, there is no clarity on who is counted as vulnerable and therefore bears the risk of arbitrariness of treatment. Furthermore, as the notion of priority treatment or attention is not further specified it may, in contrast to the concept of ‘special measures’ specified by international human rights instruments which have to fulfill a well-defined list of criteria, also lead to arbitrary preference of persons and groups, which under international law could also be considered as unlawful discrimination.^21^

The analysis showed that the frame of vulnerability mobilizes narratives that are consistent with the meaning of the word (‘wound’). Individuals and groups labeled as vulnerable are described as suffering, as those who are afflicted by harm, adversity, distress, diseases, malnutrition, violence, abuses, exploitation, and many other hardships. The many adversities are often phrased as being a characteristic or essence of these persons or groups. The vulnerability framing is furthermore based on a problematic and hierarchic association of the helpless, weak, wounded victim who lacks ability and agency and who has special needs and requires protection. Thus, the vulnerability frame makes use of a victim narrative which privileges a specific reductionist conception of a human being, which is not able to ‘accommodate a multi-layered experience’ [130, p. 6]. The repeated labeling of specific individuals and groups as vulnerable attaches all the problematic narratives that are associated with the metaphor of a wound to these individuals and groups. Rein and Schön have pointed out that ‘[f]rames try to “hitch on” to norms which resonate broader culture themes in society. This helps to explain the power that some frames exert within a policy arena.’[47, p. 89] This, on the one hand, helps us to understand, that it is no coincidence, that persons framed as vulnerable are predominately women, children, persons of color, disabled, the poor, underdeveloped, etc. as these narratives resonate well with stereotypical conceptualizations of gender, race and other inequality structures that are prevalent in the society and profoundly inscribed in the social, economic, and political order. On the other hand, it also helps us to understand why the concept of vulnerability is particularly popular in the human rights context as the ‘victim in need of protection’ rhetoric is deeply embedded in human rights discourses [32–34, 122, 129].

Understanding vulnerability as a frame and acknowledging its linguistic implications also raises the question of whether the concept invokes sexist or racist narratives. Concerning, for example, the latter, it has been pointed out that language and discourses play an important role in the production and reproduction of racism [130–136]. ‘[R]acist attitudes and beliefs are produced and promoted by means of discourse’ [136, p. 476] However, studies also revealed that racist discursive structure ‘only seldom appears as open racism’, instead they use ‘codes and metaphors that camouflage the racist message.’ [131, p. 2] It has been indicated before that the fact that the concept of vulnerability is based on a ‘somatic’ metaphor is crucial when it comes to understanding the limitation of the concept. Racist, sexist, and other discriminatory discourses have a long history of relying on somatic and natural metaphors that indicate weaknesses, inabilities, bodily differences, threats, helplessness, and many other grievances. Therefore, it is very troubling to use such concepts because they may contribute to the perpetuation of racist, sexist, and other stereotypic, stigmatic, and discriminatory narratives. In addition, in international human rights law there is a clear understanding that gendered, racist, ableist, age-based, and other stereotypes, prejudices, and stigma are incompatible with human rights obligations and must be addressed in order to move toward the goal of substantive equality [102, 103, 106, 107, 137, 138].^22^

Frame theory indicates that it is not possible to just ‘free’ a particular frame from its troubling meaning and associations as they refer to the very essence of the concept. Instead, it would be necessary to choose different concepts, which enable other and more empowering narratives, which also conceptually include human relationships and, thus, are able to grasp power relations. It is also not sufficient to just exchange single concepts. Instead, it would be necessary to create a different human rights discourse that enables concepts, which accommodate multi-layered and multi-faceted aspects and experiences of human beings including structural and relational aspects of society and politics.

## Footnotes

1 See also contributions to the Special Issue on vulnerability in this Journal, for example [2–6].

2 See also [13, 20, 23]

3 State and other reports submitted in the context of reporting procedures such as the Universal Periodic Review or in the context of specialized human rights treaties were not considered in this study.

4 For an overview, see https://www.ohchr.org/en/climate-change/integrating-human-rights-unfccc (last visited May 9, 2023).

5 At the time of writing the last draft of this article, in August 2023, a final version of the General comment was not yet available.

6 See also similar arguments in [79, 97]

7 See also [78, p. 17].

8 There are many examples of this practice of framing specific problems, issues, or social categories as the vulnerability of affected groups or individuals. Other examples of this practice are the lack of resources in order to choose migration as an adaptation strategy [79, 100, 79

9 However, in the process of reviewing many different documents only one UN document could be found, which was outside the scope of this study as it had no profound focus on climate change or was not originating from a human rights body and, thus, was not selected for this analysis, explicitly mention men and boys as being potentially vulnerable: the 2019 The *Global Compact for Safe, Orderly and Regular Migration*.

10 See, for example, [102–108]

11 See also [77, Para. 53].

12 See, for example, [108, 113].

13 See, for example, [74, 75, 77]

14 This point has been raised by several scholars. It has been argued, for example, that vulnerability is an ‘ontological condition’ [1, p. 9], and that vulnerability approaches tend ‘to attribute this condition to something about them, their intrinsic characteristics or properties’ [16, p. 201]. Furthermore, it has been pointed out that the ‘vulnerable body pertains to that which is given, to that which is immediate and never to that which is elaborated’ [30, p. 155] or that vulnerability approaches ‘pathologise social disadvantage’ [27, p. 677].

15 As mentioned in the introductory section, frame analysis directs attention to the choice of concepts or, in the case of vulnerability, metaphorical concepts, and their implications. The attention of the concept of vulnerability focuses on the wound and persons and groups who are wounded and who are suffering. Thus, the concept does not direct the attention to a relationship between persons as, for example, the word discrimination does, which means exclusion or segregation.

16 There is ample evidence as to who is causing and driving climate change, see for example reports published by the Intergovernmental Panel on Climate Change such as [118]

17 See, for example, [70, 119]

18 See also [120, Para. 7].

19 See, for example, [121–127]

20 See, for example, [128]

21 See, for example, definition of discrimination in Article 1 of the International Convention on the Elimination of All Forms of Discrimination or Article 1 of the Convention on the Elimination of All Forms of Discrimination against Women.

22 CEDAW, for example, explicitly obliges State Parties in Art. 5 to take all appropriate measures ‘modify the social and cultural patterns of conduct of men and women, with a view to achieving the elimination of prejudices and customary and all other practices which are based on the idea of the inferiority or the superiority of either of the sexes or on stereotyped roles for men and women’ [103, Art. 5]. The CERD Committee recommends State Parties to ‘[t]ake resolute action to counter any tendency to target, stigmatize, stereotype or profile, on the basis of race, colour, descent, and national or ethnic origin’ [138].
